# Localized Quantitative Analysis of Polymeric Films through Laser Ablation–Inductively Coupled Plasma Mass Spectrometry

**DOI:** 10.3390/polym13030345

**Published:** 2021-01-22

**Authors:** Ángela Villaseñor, Raquel Sánchez, Marina Boccongelli, José-Luis Todolí

**Affiliations:** 1 Department of Analytical Chemistry, Nutrition and Food Sciences, University of Alicante, P.O. Box 99, 03080 Alicante, Spain; angela.vm@ua.es (Á.V.); r.sanchez@ua.es (R.S.); 2 Total Research & Technology Feluy, Zone Industrielle C, B-7181 Feluy, Belgium; marina.boccongelli@total.com

**Keywords:** ICP-MS, laser ablation, polymers, packaging films, spatially resolved analysis

## Abstract

The present work shows, for the first time, the application of laser ablation connected to inductively coupled plasma mass spectrometry (LA-ICP-MS) to the localized quantitative analysis of inclusions in polymeric industrial films. The multielemental mapping capabilities of LA-ICP-MS has allowed to chemically examine unique defects appeared during the plastic processing. This analytical tool is perfectly suited to detect elements such as Al, Mg, Zr, Ti, Cr, P, Pb, Sb, Zn, and Si in those inclusions. A method for multielemental quantitative analysis of these defects has been developed in the present work. The profiling for more than 100 different defects in three samples has demonstrated that more than 50% of these inclusions contain aggregates of some of the aforementioned elements. Therefore, the distribution of elements used as additives or present in catalysts must be carefully controlled during the production of polymeric films in order to avoid degradation in their performance.

## 1. Introduction

Laser ablation adapted to inductively coupled plasma mass spectrometry (LA-ICP-MS) is an established analytical tool employed for measuring amounts (or isotope ratios) of elements in solid samples with a spatial distribution in the micrometer range [1,2]. Compared with other spatially resolved techniques, such as secondary ion mass spectrometry (SIMS), sputtered neutral mass spectrometry (SNMS) and X-ray fluorescence (XRF), LA-ICP-MS affords higher sensitivities and lower limits of detection and allows determining both major and trace elements in the same sample [3].

LA spatial analysis was first demonstrated for biological samples by Feldmann et al. [4] and is now typically referred to as bioimaging or mass spectrometry imaging (MSI) [5,6]. This kind of assays can be performed in two different ways: (i) in the spot ablation, the micro-sampling of the material is produced from a single point; (ii) when applying rastering or line/multiline ablation, elemental/isotopic spatial distribution is assessed over a surface of a sample [7]. The major difference between both ablation methods is that in the former one a rapid decrease in signal intensity is observed whereas for the latter one, the signal is higher and more steady. The spatial resolution during surface mapping depends on the spot diameter, whereas the number of laser pulses fired determines the amount sampled.

Sample preparation for spatially resolved analysis through LA-ICP-MS depends on the material to be analyzed. Thus, simple slicing sectioning can be applied, for instance. However, the procedure for preparing samples can be somewhat complex. In the case of sediment analysis, freeze coring is a good approach as it has been demonstrated to maintain integrity of sediment layering at a centimeter scale [8].

In localized analysis, the design and dimensions of the ablation cell are critical and preclude the spatial resolution of the method. The use of an ablation cell with very small volume having a very short washout time is recommended. Indeed, the sample can be directly ablated into the transport tube, thus giving rise to the so-called short tube ablation cell [9]. In a different approach, a Laurin two volume ablation cell is proposed. This is a funnel-shaped ablation cell with a 1–2 cm^3^ inner volume and a hole at the bottom. This funnel, inserted into a 380 cm^3^ box cell, is above the sample and remains stationary whereas the sample box is in movement [10]. The two volume ablation chamber provides a washout time typically shorter than 1.5 s.

Among the numerous applications of LA-ICP-MS for the localized elemental analysis, one can mention mapping of annual rings of trees [11], bark and bark pockets [12,13], mussel shells [14], leaves [15], rice [16], lake sediments [17] and wheat [18] grains. The use of LA-ICP-MS as a surface analytical technique for the determination of lateral element distribution has also been described for ceramic layers [19] and alloys [20].

LA-ICP-MS and LA-ICP-OES have also been applied to the quantitative elemental bulk analysis of polymers [21], including polymeric films [22]. It is worth mentioning that, during the production process of polymeric films, heterogeneities, or defects, may appear. These inclusions can have a different elemental composition according to their preparation, catalytic processes and/or may be originated as a result of a different polymerization process as compared to the rest of the film. In the particular case of polymeric films these defects may lead to a degradation of their performance in terms of physical, including barrier, properties. Uneven spatial distribution of metals, used as additives or migrated from catalysts, can be in the origin of the generation of these inclusions.

To the best of our knowledge there are not systematic studies related with the application of LA-ICP-MS to the localized analysis of polymeric industrial films. The goal of the present study was, thus, to develop a LA-ICP-MS method for the quantitative multielemental analysis of defects on this kind of materials. The elemental profile within a given inclusion, together with the application of a calibration method that could compensate for changes in the sample composition was thus achieved.

## 2. Materials and Methods

### 2.1. Samples

Polymeric films supplied by Total Research and Technology, Feluy, Belgium, were analyzed. Five of them corresponded to transparent polypropylene casts and the last one was a less flexible black film. The polymeric films were prepared from polymer powder transformed into pellets by extrusion. These pellets were extruded through a flat die under specific conditions and a film was generated. Sample preparation simply consisted of a propan-2-ol washing and drying step prior to their analysis. According to the sizes of the defect and the laser shot diameter, only one to six scan lines were required to ablate it.

The net intensities of the different analytes found in the inclusions were registered as a function of time thus giving rise to signal lateral profiles. The signal intensity could be plotted versus the position on the film using the scan rate to transform time into length.

In order to apply the so-called external dried droplet calibration approach (E-DDCA), the sample ablated mass per pulse had to be measured. To obtain this parameter, the sample was weighed with a closed Mettler Toledo micro-balance (precision of ± 1 µg).

### 2.2. Laser Ablation System

A Nd:YAG solid state laser ablation system, CETAC LSX-213 G2+ (CETAC, Omaha, NE, USA) was used. This laser operated at 213 nm under Q-switched mode. The instrument contained the following parts:

- The laser cabinet including the active laser medium, all optics apertures, the lighting units, a charge-coupled device camera (CCD) that allowed real-time observation of the ablation process and the ablation cell placed on an adjustable XYZ platform.
- The cooler or power supply that circulated water through the laser head and provided the power necessary for the laser.
- Computer that controlled the sample position, laser firing, camera options, sample cell illumination and gas flow rate by means of the Digilaz^TM^ G2 sotfware.

The ablation process took place inside the two-volume HelEx Sample Cell. This cell was square-shaped with dimensions of up to 20 × 20 cm. It presented a cone placed over the sample above the ablated region, which contained an inlet for helium, called He1, to remove quickly the aerosol from the sample surface. The helium flowing from the ablation chamber into the cone, transporting the ablated material into the ICP transfer tube was called He2. Therefore, two separate He carrier gas flow inputs were used, leading to uniform signal responses (<2% RSD) throughout the sampling area. As a conclusion, three gas streams were used; He2, He1, and an argon stream, that delivered the particles leaving the ablation cell to the plasma through a 40 cm length transfer tube. Table 1 summarizes the LA operating conditions.

## 3. Results

Unique inclusions in polymeric films either visible to the human eye or only visible upon microscopic examination were previously detected and selected for their further analysis. Results are mainly presented for three representative samples: film # 1, a transparent and flexible sample, for which a total of 40 inclusions were characterized; film # 2, of the same kind as # 1, with 30 defects detected and analyzed; and, film # 3, a black non-flexible sheet, containing visible irregularities on its surface. In this latter case, a total of 25 of these defects were laser ablated.

### 3.1. Results for Film # 1

**Figure 1:**
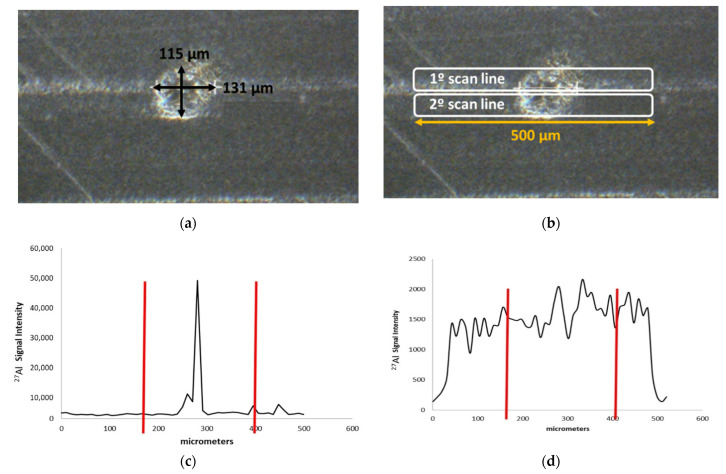
Dimensions of defect # 2 found on film # 1 (**a**); laser scan lines (**b**); ^27^Al signal variation versus laser position for the first (**c**) and (**d**) second scan line, red vertical lines show the limits of the inclusion.

Figure 1 shows the dimensions of one of the defects found on film # 1, ablated using two 50 μm wide scan lines separated by a distance of 12 μm. As it may be observed, the ^27^Al ICP-MS signal peaked at the center of the inclusion (Figure 1c). This huge spike was observed only for the first scan line. Taking into account the variation of the signal across it, two overlapped ^27^Al spikes were found: a small one having a width of around 50 μm and the most intense peak with a size of roughly 250 μm. Therefore, from Figure 1c, it could be concluded that the examined defect contained two Al accumulations or particles that were included or deposited during the film production process.

The ^27^Al signal coming from the second scan line, instead, did not change when the inclusion was ablated (see Figure 1d), i.e., the lower part of the inclusion had the same aluminum composition as the rest of the film. These former results revealed that the inclusions themselves were chemically heterogeneous in terms of Al concentration. The multielemental capability of the ICP-MS allowed us registering the analytical signals for various isotopes from scan line 1 in Figure 1. It was verified that the signals corresponding to the rest of elements providing ionic counting distinguishable from the background (Zr, Cr, Mg, Ti, Zn, Si, and Pb) did not change as the inclusion in Figure 1 was ablated. In other words, the distribution of these elements was uniform in the polymeric film regardless of the presence of inclusions.

Regarding the carbon signal, it was significantly higher when the laser was on than when measured the background caused by the atmospheric CO_2_ diffusing towards the plasma central channel. The results demonstrated that the ^13^C intensity did not change as the inclusion was ablated with respect to the main uniform film material. This fact led to two conclusions: (i) the signal increment observed for Al was not due to a modification in the extent of the interaction between the laser and the sample surface; and, (ii) the defect was not due to a different polymerization on that point. Note that a change in the polymer characteristics would have modified the carbon ablation yield and, hence, the ^13^C ICP-MS signal.

**Figure 2:**
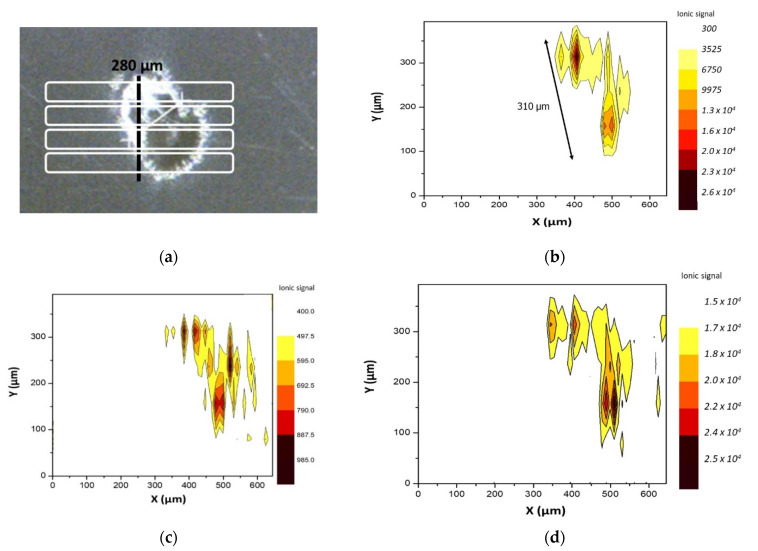
Defect found on film # 1 requiring multiple rasters. (**a**) laser line scans required for complete defect sampling and (**b**) ^27^Al; (**c**) ^98^Zr and (**d**) ^24^Mg signal distributions within it.

Big inclusions containing more than one element were also found on film # 1 (Figure 2a) requiring at least four 50 μm width scan lines for full defect coverage. In order to evaluate the signal spatial evolution, a contour plot was obtained. For the sake of clarity, the y-axis scale was expanded. Figure 2b shows the distribution of the ^27^Al signal throughout the ablated area. It was clearly observed that when the inclusion was ablated, the Al signal significantly increased. As it may be observed, the dimensions of the plot where the aluminum signal was distinguishable agreed with the actual dimensions of the inclusion. In this particular case, more elements were detected. Because various nuclides were monitored for each scan line, it was clearly verified that the trend discussed for Al was also found for Zr (Figure 2c) and Mg (Figure 2d). The rest of studied elements (Cr, Ti, Zn, and Pb) did not yield distinguishable signals with respect to the main film.

By a closer inspection of the elemental distribution in Figure 2, it was observed that four main multielemental accumulations were present in this irregularly shaped inclusion.

The same treatment as that previously described was performed on a total of 40 defects on film # 1 and the signals for the analytes included in Table 2 were obtained as a function of the ablation position on the sample surface. The maximum signal intensities obtained for the elements present in these inclusions are summarized in Figure S1. Figure 1a shows the results found for ^27^Al and ^24^Mg that were present in most of the inclusions. In Figure 1b. the remaining tested nuclides (i.e., ^98^Zr, ^66^Zn, ^52^Cr, ^47^Ti, and ^208^Pb) are considered. It is interesting to notice that only 13 out of 40 defects provided appreciable elemental ICP-MS signals (Figure S1).

### 3.2. Results for Film # 2

**Figure 3:**
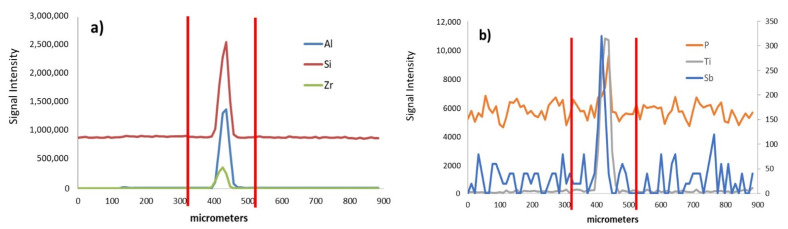
(**a**) Al, Si, and Zr (**b**) P, Ti, and Sb signal intensities versus the length of the first scan line used to ablate the inclusion # 1 present in film # 2. Signals for Sb are shown in the right y-axis.

For this second film, an inclusion with a diameter of 100 μm (defect # 1) was ablated using a laser beam dimeter of 50 μm. Thus, two scan lines were required to ablate it completely. When the first scan line was registered, the signals for Al, Si, Zr, P, Ti, and Sb increased as the inclusion was ablated (Figure 3). In contrast, for the second scan line on the same location, thus corresponding to the bottom of the inclusion, the signal intensities were not modified as the defect was ablated (data not shown). Therefore, these elements were accumulated in the uppermost part of the inclusion. The capability of LA for spatial resolution studies allowed to conclude that the elements found in Figure 3 were present in the same location (i.e., from 400 to 450 μm) thus revealing the presence of a multielemental 50 μm diameter particle.

**Figure 4:**
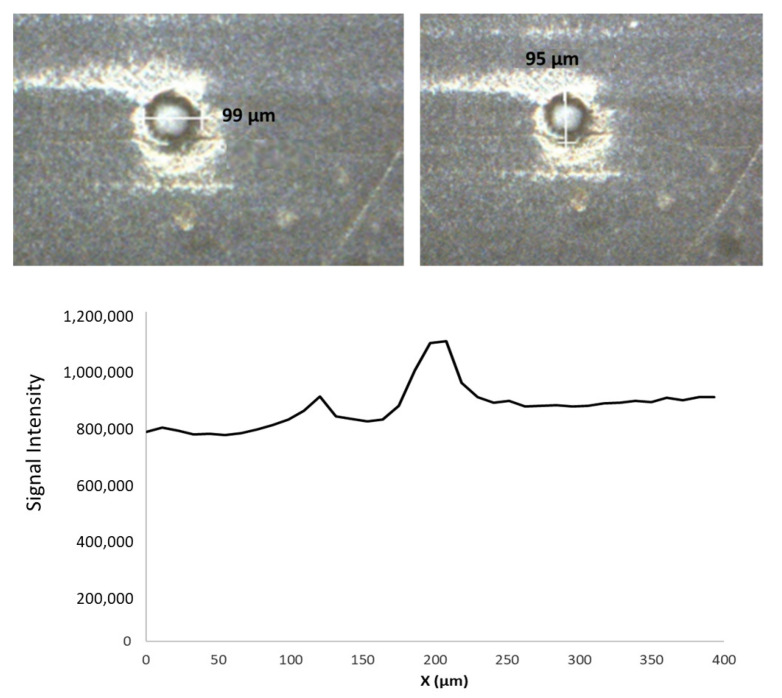
Picture and dimensions (up) and ^13^C signal intensities versus the length of the first scan line (down) used to ablate the inclusion # 16 present in film # 2.

The behavior for carbon ICP-MS signal was dependent on the inclusion sampled. Interestingly, in the case of defect # 16, the ^13^C ionic intensity increased by a 20% as it was ablated (Figure 4). For this situation, it could be indicated that, when the laser interacted with the inclusion bottom, it was slightly defocused. This might cause an increase in the sample illuminated area and, hence, in the ablated mass. An alternative explanation would be that the polymer nature in the defect could be different as compared to the film and, thus, the ablation yield increased.

Figure S2 summarizes the maximum signal intensities obtained for the elements present in the inclusions found in film # 2. For this sample, it was observed that Si and Al provided the highest signal intensities in most of the inclusions. Note that Si was not found in film # 1. Meanwhile, Ti afforded high signal intensity only when defect # 16 was ablated and Cr and Zr prevailed in the case of defect # 18. Figure 2b considers elements supplying low ICP-MS signals such as Mg, P, Sb, Zn, and Pb. Among all the studied defects, it appeared that inclusion # 18 was the most polluted one. Contrarily to the situation previously discussed (film # 1), the majority of the defects (20 out of 30) in film # 2 contained metals.

### 3.3. Results for Film # 3

Figure S3 shows the appearance of some inclusions present in film # 3. The inclusions were darker than the polymeric film and their surfaces were irregular.

**Figure 5:**
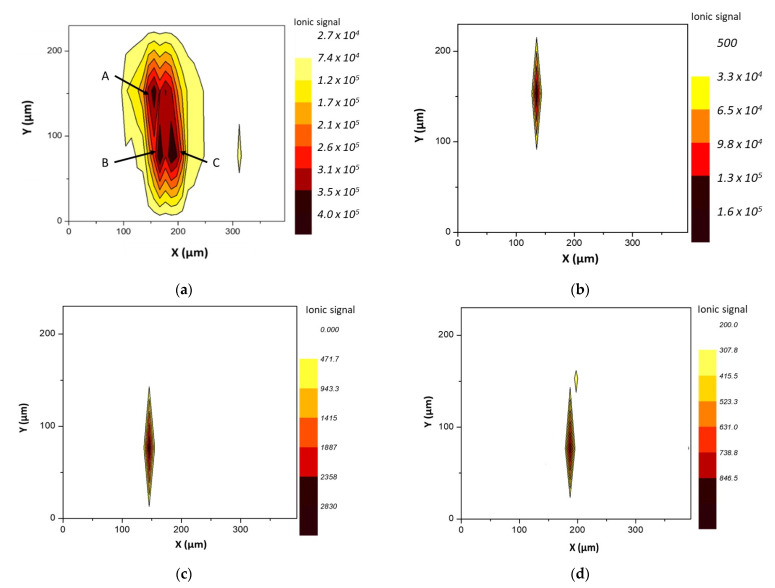
Contour plots for (**a**) ^27^Al (**b**) ^52^Cr, (**c**) ^98^Zr, and (**d**) ^208^Pb corresponding to the 4 scan lines performed on defect #1 9 of film # 3.

The multielemental signal intensity was registered for inclusion # 19. Figure 5a shows that aluminum was widely distributed throughout the inclusion, being more concentrated at its center. Interestingly, the dimensions of the area containing this element were larger than those for the inclusion visually taken. This fact revealed a progressive diffusion of aluminum from the default to the sample bulk. From the data in Figure 5, it was concluded that three-four particles were present in this default having a high content of Al. Also interesting was to compare these data with the plots for other elements, because it was clearly concluded that Cr coexisted with Al in deposit A (Figure 5a,b). Meanwhile, Zr and Pb were mainly present in particles B (Figure 5a,c) and C (Figure 5a,c), respectively. These observations, led to the conclusion that Al particles containing other contaminants were the responsible for these defects on the polymer finally obtained.

## 4. Discussion

### 4.1. Development of a Localized Quantitative Analysis Method

Although the experiments suggested that, for most of the cases, the matrix of the inclusion did not appreciably modify the ablation process, a study was undertaken to fully characterize the impact of the matrix on the ICP-MS signal, i.e., matrix effect. Droplets of an aqueous multielemental standard (20 mg/kg) were deposited on films # 2, # 3, and # 4. After solvent evaporation, the solid residues were completely ablated. The elements studied (i.e., Cd, Pb, and Cr) were not present in the considered films and, thus, their signals came exclusively from the solid residues. Figure S4 reveals that the signals taken were more than 20% different (see boxes on these figures) depending on the element studied. For example, for ^111^Cd, the signal intensities were dependent on the films analyzed (Figure S4a). The ICP-MS ionic signals observed for ^208^Pb, in turn (Figure S4b), were similar for the solid residues deposited on films # 2 and # 3 but different from that obtained in film # 4. Only for ^52^Cr, the signals corresponding to the solid residues were similar independently of the film considered (Figure S4c). In order to compensate for these changes in ICP-MS intensities as a function of the matrix of the film studied, ^45^Sc was selected as an internal standard. This element was initially present in the multielemental solution and its signal was taken together with those for the remaining analytes. The found results suggested that the matrix effect either remained or was even intensified.

It was concluded that a method in which small volumes of a set of standards were deposited on the film main surface as aqueous solutions could be a suitable means for obtaining quantitative information about the defects elemental composition. Thus they could eliminate the impact of the sample matrix composition on the ICP-MS sensitivity.

Once the standards were settled, water was evaporated at room temperature and the solid residues were subsequently ablated, the signals were measured and the calibration line was finally obtained. Analyte concentration was calculated by interpolation of the signal found for the inclusion in the calibration line (see Figure S5). Thus, this method was similar to a matrix matching external calibration procedure and received the name of external dried droplet calibration approach (E-DDCA).

In order to apply the E-DDCA method, a given film was ablated using a known number of laser pulses (114,000 pulses) and a laser spot size of 50 μm. The film was weighed before and after the ablation and the difference between the initial and final weight (*M_s_*) was calculated. In the case of film # 1, for instance, this mass difference was 0.226 mg. Once the number of pulses, *N_p_*, and the ablated sample mass, *M_s_*, were known, the sample ablated mass per pulse, *M_p_*, was calculated according to Equation (1).

(1) $$M_{p}=\frac{M_{s}}{N_{p}}=\frac{0.226\;\mathrm{mg}}{11400\;\mathrm{pulses}}=2\;\mathrm{ng}/\mathrm{pulses}$$

The next step was to deposit 1 μL of different solutions containing aluminum (together with 22 additional analytes) from 0 to 20 mg/L on the film by means of an automatic pipette. Methylene blue was also present in the solutions in order to visually focus the laser beam on the right area of the films with the help of the instrument camera.

A high percentage of the solid residue deposited on film was ablated. This percentage, *S_A_*, was calculated according to

(2) $$S_{A}=\frac{ablated\;area}{total\;residue\;area}=\frac{\underset{N_{r}}{\sum }L_{r}\cdot Ø}{\underset{N_{r}}{\sum }L_{r}\cdot Ø+\underset{N_{r}-1}{\sum }L_{w}\cdot S_{L}}100$$

where *Ø* was the laser beam diameter, *L_r_* the length of each particular line scan, *N_r_* the number of line scans used to ablate the dried droplets, *S_L_* the distance between line scans, and *L_w_* the length of the non-ablated sample surface.

The percentage of each solid residue ablated for the residues originated from standard solutions of 0, 5, and 20 mg/L were 82%, 83%, and 80%, respectively. Once *S_A_* was known, the mass of aluminum ablated from each solid residue was calculated according to Equation (3). In the case of the solution of 5 mg/Kg, this mass was 4 ng.

(3) $$m_{A}=C*V*S_{A}=5\frac{\mathrm{mg}\;of\;AL}{\mathrm{Kg}}*1\;\mu g*10^{-6}\frac{\mathrm{Kg}}{\mu g}*0.82=4\;\mathrm{ng}\;of\;Al\;ablated$$

From the number of pulses applied to ablate each solid residue (8850 in the case of the solid residue resulting from the 5 mg/kg droplet) it was possible to obtain the aluminum ablated mass per pulse from each solid residue, being 0.0005 ng in the case of the 5 mg/kg droplet.

It is interesting to note that for quantitative purposes, the sum of intensities obtained for a given solid residue was divided by the number of pulses required to ablate it. Therefore, the calibration line was obtained by plotting the sum of intensities for each solid residue per pulse, *I_i_*/*N_p_*, versus the aluminum ablated mass per pulse, *m_A_*/*N_p_*.

In order to transform the aluminum signal intensity per pulse into concentration, the equation corresponding to the calibration line was used. The calibration line related the signal intensity per pulse versus the Al ablated mass per pulse. Finally, the concentration of aluminum in the sample (*C_A_^s^*) was calculated according to Equation (4) for each point on the scan line used to ablate the inclusion.

(4) $$C_{A}^{S}=\frac{m_{A}}{M_{p}}$$

### 4.2. Application of the E-DDCA Method

The E-DDCA was applied to the defects found in two different films: # 1 and # 2. The methodology followed is exemplified, as supplementary material, for the determination of Al in the defect # 2 present in film # 1. Once the calibration line was obtained, the signal intensity was registered along a given defect (Figure S6a) and the measured signals were individually divided by the number of pulses used. Then, the resulting data were plotted versus the length of the scan line (Figure S6b). By interpolation in the calibration line, previously obtained, ablated Al mass per laser pulse was obtained (Figure S6c). The concentration in the sample was finally obtained by dividing each one of the points in Figure S6c by M_p_ previously measured (Figure S6d).

**Figure 6:**
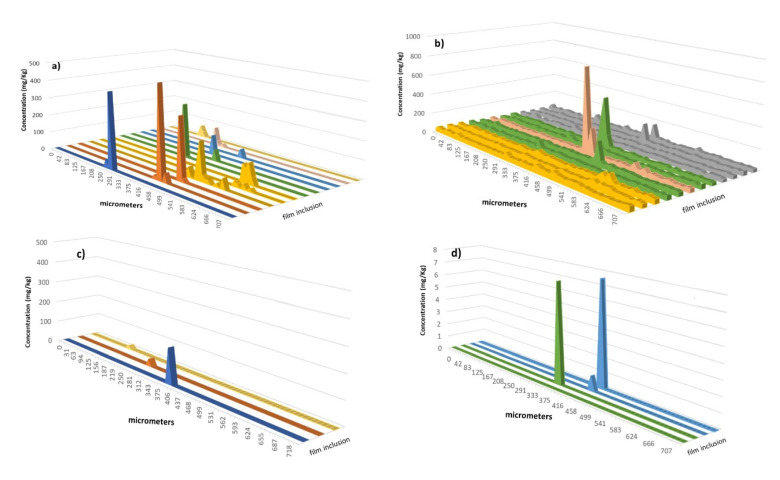
Concentration profiles of (**a**) Al, (**b**) Mg, (**c**) Ti, and (**d**) Cr along the scan line used to ablate the defects in film # 1. Only the inclusions yielding ICP-MS signals for the monitored elements are included.

**Figure 7:**
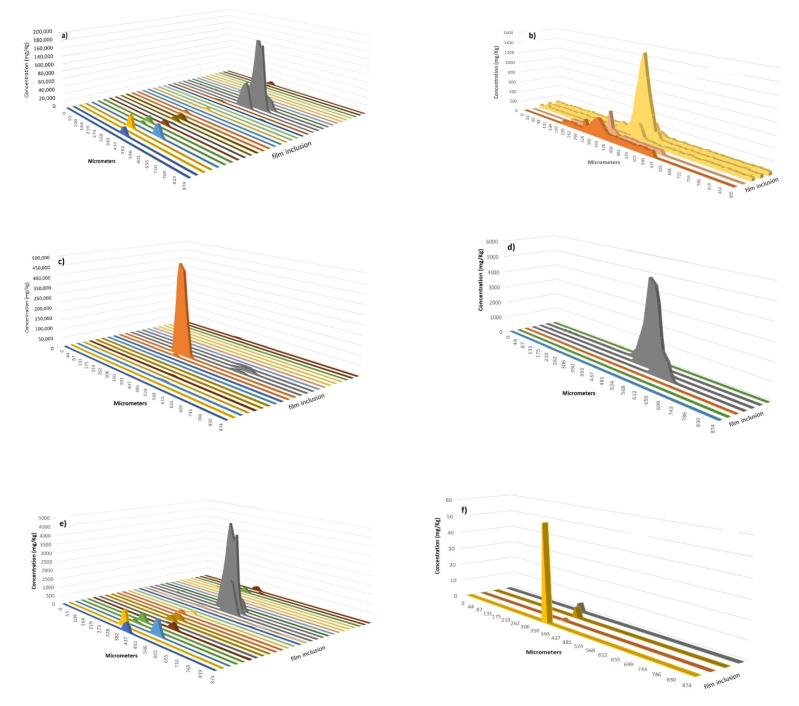
Concentration profiles of (**a**) Al, (**b**) Mg, (**c**) Ti, (**d**) Cr, (**e**) Zr, and (**f**) Pb along the scan line used to ablate the defects in the film #2. Only the inclusions yielding ICP-MS signals for the monitored elements are included.

Figure 6 and Figure 7 show the concentration of the different elements obtained along the scan lines on different inclusions in films # 1 and # 2. First, it was observed that Al and Mg were the elements most often found in the inclusions studied. Their local concentrations were fairly high reaching values of from 10^2.^–10^5^ milligrams per kilogram. This fact revealed a significant pollution at particular locations on the polymer surface.

Comparing the inclusions found on both films, it was concluded that the number and concentration of elements present were significantly affected by the sample considered. In general terms, the inclusions in film # 2 were enriched in elements with respect to film # 1. Thus, aluminum concentration was higher in film # 2 than for film # 1. In the former situation (Figure 7a), an inclusion with 160,000 mg of Al per kg of polymer was found whereas, for several defects, the concentration of this element was on the order of thousands of mg/kg. Note that the Al concentration in the defects of film # 1 was below 500 mg/kg (Figure 6a). As regards mg, this element was present at high concentrations in an inclusion investigated for film # 2 (Figure 7b). However, for most of the situations, the defaults contained similar Mg contents regardless the sample considered. TiO2 is commonly added to polymers and some inclusions contained Ti, especially for a defect in film # 2 (Figure 7c) for which a huge concentration in this element was found (i.e., 45% w/w). In contrast, for film # 1, only three defects were encountered with Ti concentrations lower than 100 mg/kg (Figure 6c). Another element detected in several situations in both films was Cr and, although it was present as a trace element in film # 1, (i.e., concentrations of 7–8 mg/kg or lower), this element was more abundant in film # 2, its maximum concentration being around 500 mg/kg. The last element registered at significant concentrations in film # 1 was Zn, whose maximum content was 180 mg/kg and was detected in just three defects. This element was not present in film # 2. Figure 7e,f provide evidence of the presence of an element used as polymerization catalyst (i.e., Zr) and a toxic element (i.e., Pb), respectively, although both elements were only found in film # 2.

An interesting trend emerged when considering the width of the signal profiles. Thus, for instance, concentration profiles for some defects in film # 2 were wider for elements such as Al and Mg than for others such as Ti, Zr, or Pb (Figure 7). These studies also could help to monitor the diffusion of some elements in polymeric matrices.

## 5. Conclusions

LA-ICP-MS in combination with a calibration methodology based on the deposition of aqueous standards on the film surface, its drying and its final ablation has demonstrated to be an excellent tool for quantitative localized analysis of inclusions in industrial polymeric films. The so-called external dry droplet calibration approach (E-DDCA) is a modified version of the DDCA successfully used for the quantitative bulk analysis of polymers such as polyethylene, polypropylene [21], as well as alumina and CoMo catalysts, commonly used in petroleum refining processes [23]. The E-DDCA shows interesting features, because it overcomes the unwanted matrix effects as the matrix for the standards is the sample itself.

Although results were only shown for three samples, experiments were also conducted for three additional transparent films, giving rise to similar observations as those explained in the present work. The determination of the elemental composition of unique inclusions in industrial polymeric films can detect localized pollution in the raw material as well as errors occurred during the production process of plastics used in packaging applications, thus modifying their mechanical properties.

## Figures and Tables

**Table 1:** Laser ablation operating conditions.

Variable	
Scan rate (μm/s)	10
Laser beam diameter (μm)	50
Spot frequency (Hz)	20
Laser shot energy (mJ)	2.7
Laser fluence (J/cm^2^)	7.15
Wavelength (nm)	213
Helium gas flow rates (mL/min)	500/300
Carrier gas (L/min) Ar added to the aerosol leaving the ablation cell	0.03

**Table 2:** ICP-MS operating conditions.

Variable	
RF power (KW)	1.55
Cell Collision (He) (mL/min)	3.0
Plasma gas flow rate (L/min)	15.0
Auxiliar gas flow rate (L/min)	0.9
Integration time/mass s^−1^	0.1
Nuclides measured	^11^B, ^12^C, ^13^C, ^27^Al, ^24^Mg, ^28^Si, ^31^P, ^40^Ar ^45^Sc ^47^Ti, ^51^V, ^52^Cr, ^55^Mn, ^56^Fe, ^59^Co, ^60^Ni, ^63^Cu, ^66^Zn ^75^As, ^81^Br, ^88^Sr, ^90^Zr, ^95^Mo, ^111^Cd, ^107^Ag, ^121^Sb, ^136^Ba,^202^Hg, ^208^Pb

## Data Availability

Data are contained within the article or supplementary material.

## Supplementary Materials

The supplementary materials are available online at https://www.mdpi.com/2073-4360/13/3/345/s1, Figure S1: ICP-MS intensities of (a) major and (b) minor elements present in the different defects found in film #1. Figure S2: ICP-MS intensities of (a) major and (b) minor elements present in the different defects found in film # 2. Figure S3: Pictures of the surface of film # 3. (a) general view and (b) detail of the defects present in the film. Figure S4: (a) 111Cd; (b) 208Pb; and (c) 52Cr ICP-MS intensities corresponding to the solid residues left after evaporation of a 20 mg/L standard. The boxes give a 20% acceptability interval. Figure S5: Scheme of the quantification methodology followed for the multielemental analysis of inclusions in polymeric films. Figure S6: ICP-MS 27Al signal versus scan line length (a), 27Al signal per pulse versus scan line length (b), Aluminum ablated mass per pulse versus the scan line length (c) and Aluminum concentration (mg/kg) versus the scan line length (d) corresponding to the defect # 2 of the film # 1.
