# Supplementary material for: Localized Quantitative Analysis of Polymeric Films through Laser Ablation–Inductively Coupled Plasma Mass Spectrometry
Source: Polymers (Basel). 2021 Jan 22;13(3):345. doi: 10.3390/polym13030345 (PMC7926387; doi:10.3390/polym13030345)
Supplement: Supplementary file 1 [file polymers-13-00345-s002.pdf]

# Localized Quantitative Analysis of Polymeric Films Through Laser Ablation–Inductively Coupled Plasma Mass Spectrometry

**Ángela Villaseñor [1], Raquel Sánchez [1], Marina Boccongelli [2], and José-Luis Todolí [1,*]**

[1] Department of Analytical Chemistry, Nutrition and Food Sciences, University of Alicante, P.O. Box 99, 03080 Alicante, Spain; angela.vm@ua.es (A.V.); r.sanchez@ua.es (R.S.)
[2] Total Research & Technology Feluy, Zone Industrielle C, B-7181 Feluy, Belgium; marina.boccongelli@total.com
* Correspondence: jose.todoli@ua.es

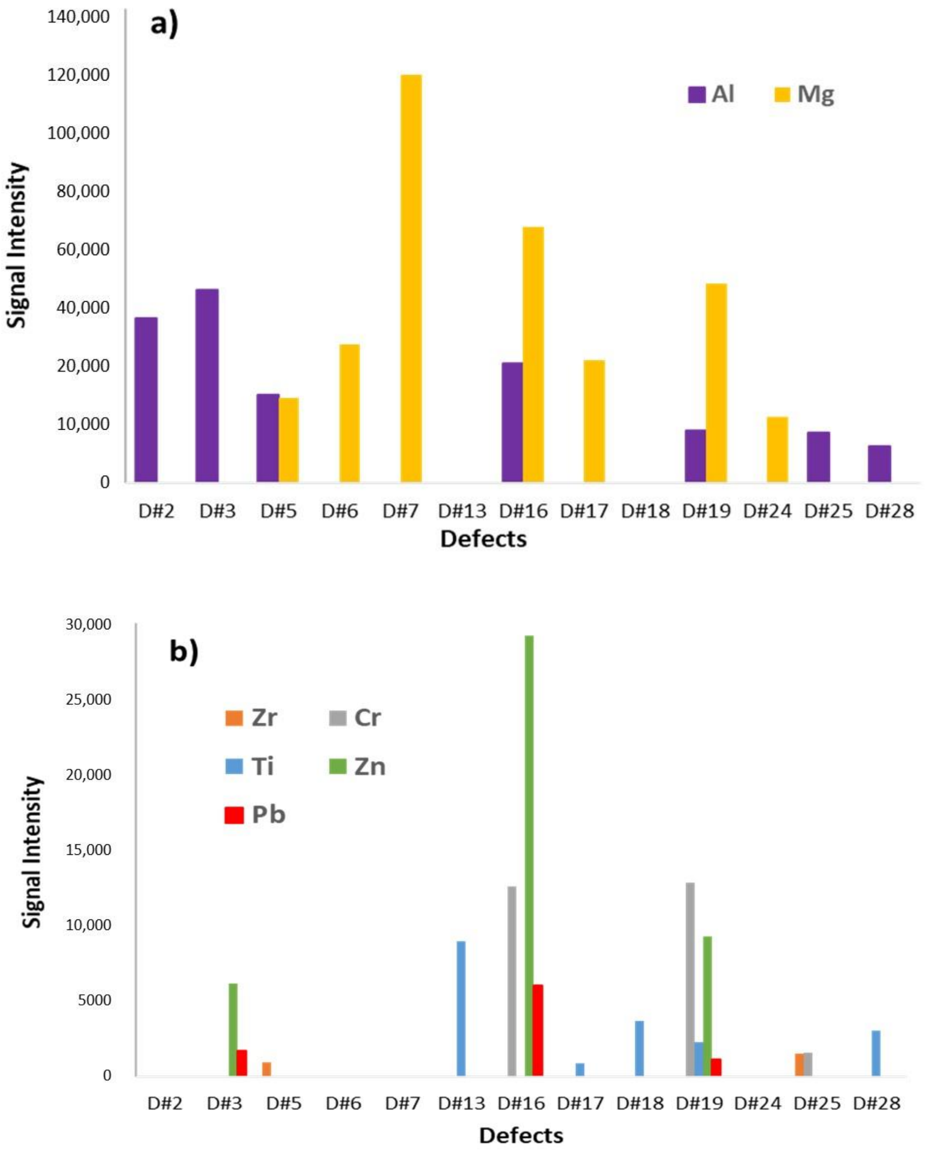


**Figure S1.** ICP-MS intensities of (**a**) major and (**b**) minor elements present in the different defects found in film #1.

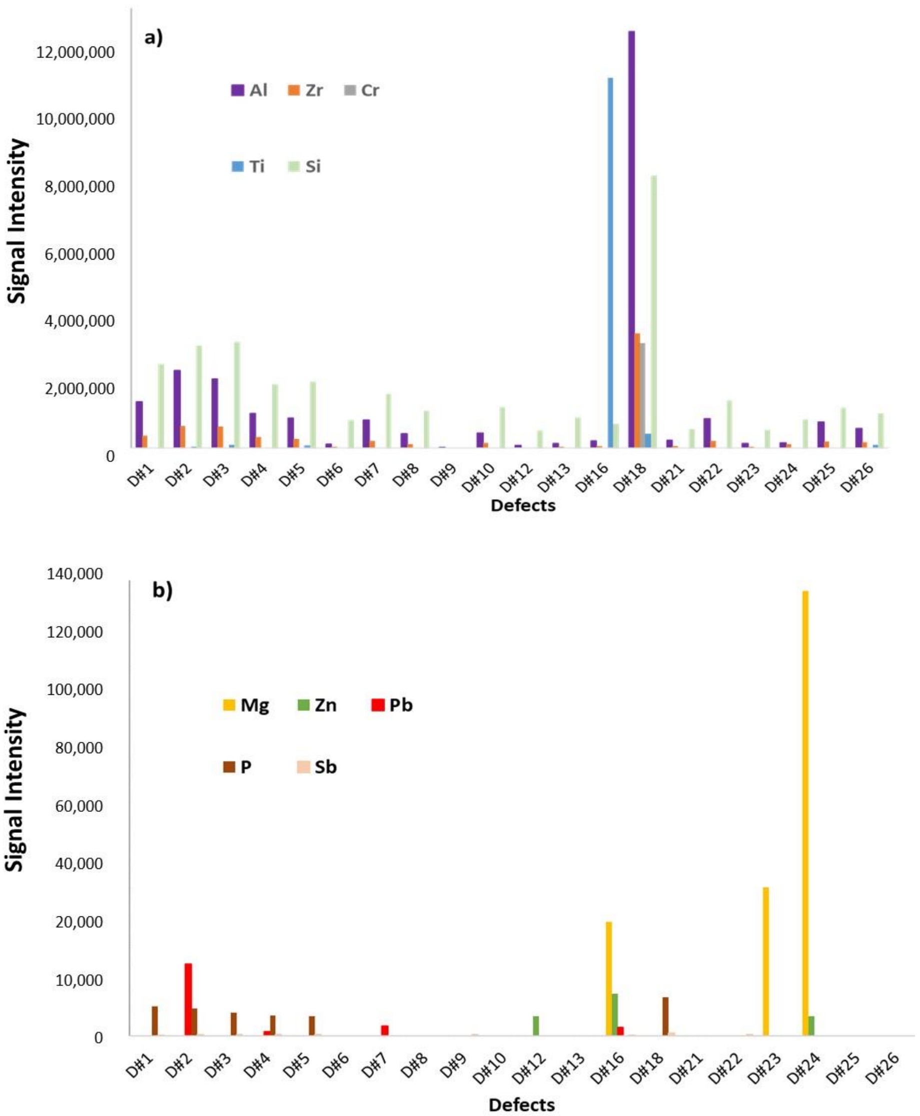


**Figure S2.** ICP-MS intensities of (**a**) major and (**b**) minor elements present in the different defects found in film # 2.

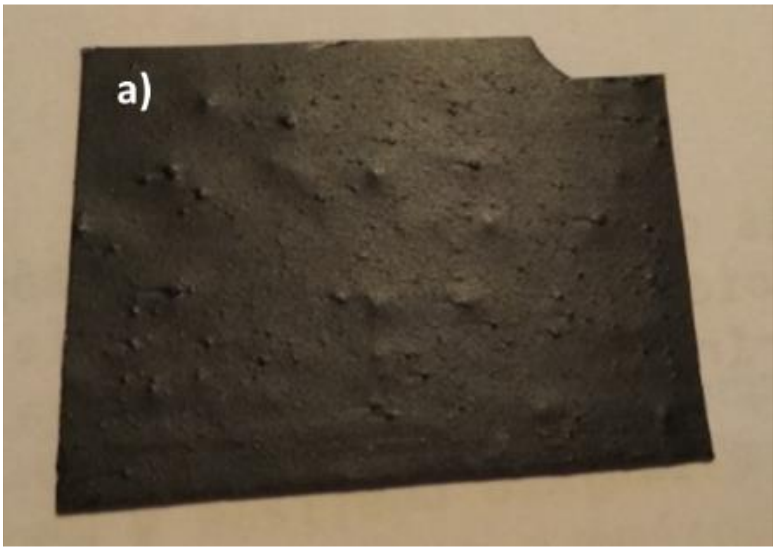


(a)

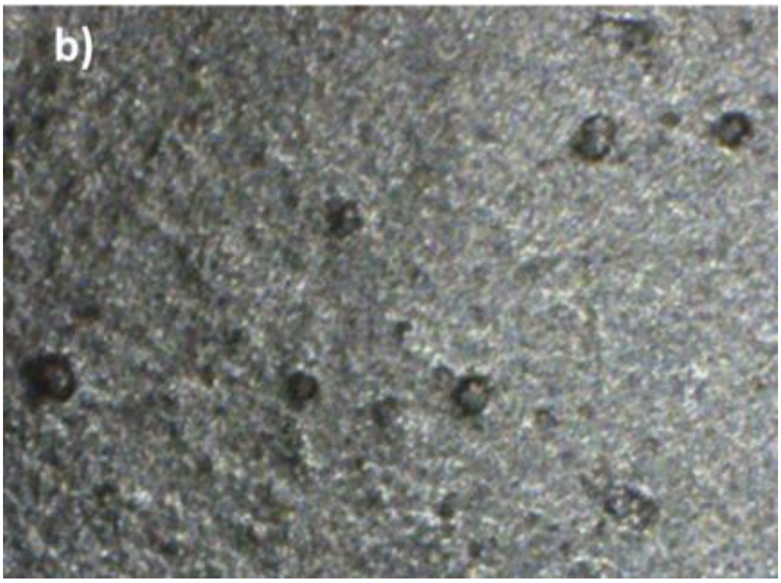


(b)

**Figure S3.** Pictures of the surface of film # 3. (**a**) general view and (**b**) detail of the defects present in the film.

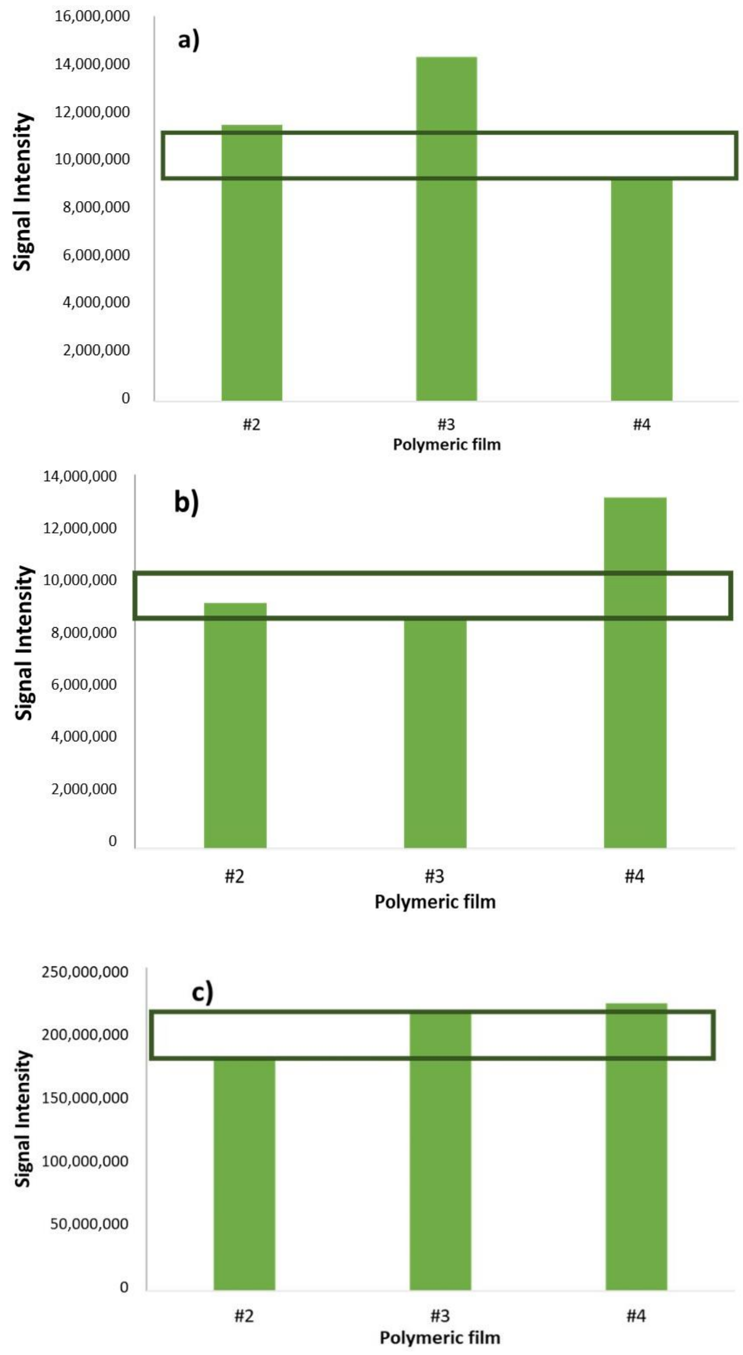


**Figure S4.** (**a**) $^{111}$Cd; (**b**) $^{208}$Pb; and (**c**) $^{52}$Cr ICP-MS intensities corresponding to the solid residues left after evaporation of a 20 mg/L standard. The boxes give a 20% acceptability interval.

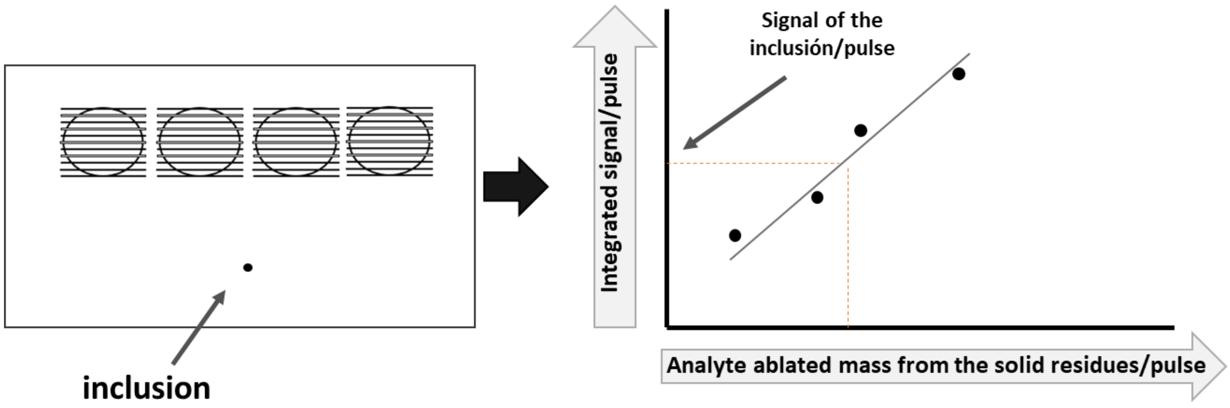


**Figure S5.** Scheme of the quantification methodology followed for the multielemental analysis of inclusions in polymeric films.

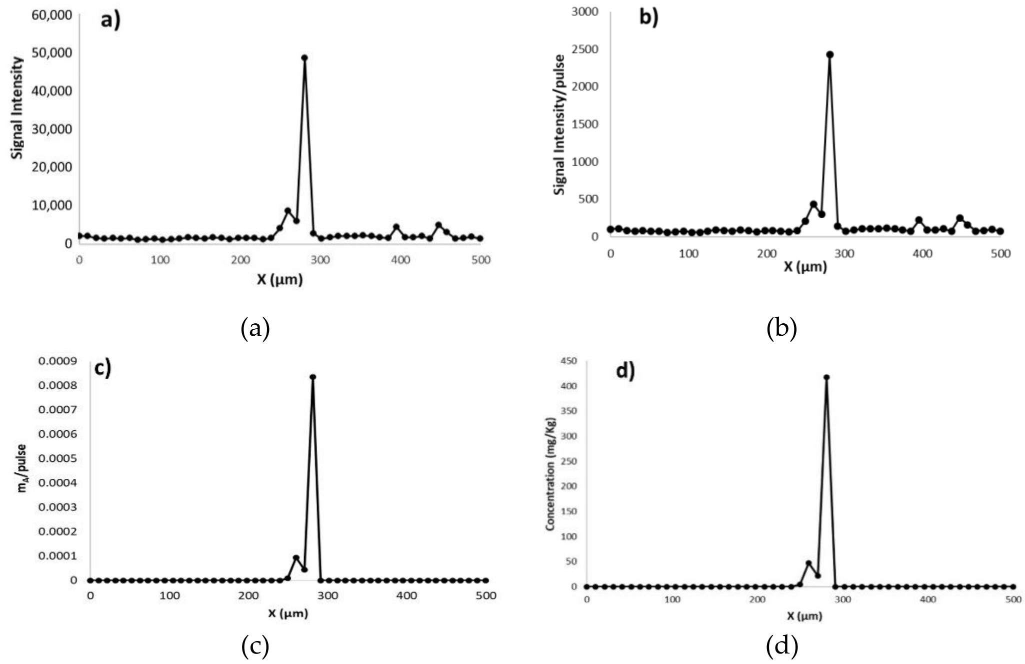


**Figure S6.** ICP-MS $^{27}$Al signal versus scan line length (**a**), $^{27}$Al signal per pulse versus scan line length (**b**), Aluminum ablated mass per pulse versus the scan line length (**c**) and Aluminum concentration (mg/kg) versus the scan line length (**d**) corresponding to the defect # 2 of the film # 1.
